# Effect of Melatonin as an Adjunct to NSPT on Periodontal and Systemic Outcomes in Patients with Type 2 Diabetes Mellitus: A Systematic Review and Meta-Analysis of RCTs

**DOI:** 10.3390/jcm15114071

**Published:** 2026-05-25

**Authors:** Thaleia Angelopoulou, Yiorgos A. Bobetsis

**Affiliations:** 1 School of Medicine, National and Kapodistrian University of Athens, 11527 Athens, Greece; thangelop@yahoo.gr; 2 Department of Periodontology, School of Dentistry, National and Kapodistrian University of Athens, 11527 Athens, Greece

**Keywords:** periodontitis, periodontal diseases, non-surgical periodontal therapy, melatonin, melatonin supplementation, type 2 diabetes mellitus, metabolic profile, HbA1c, systematic review

## Abstract

**Background/Objectives**: In recent years, a wide range of adjunctive therapies have been employed in conjunction with non-surgical periodontal therapy (NSPT) to enhance treatment outcomes. Among them, melatonin, a hormone with well-documented antioxidant, anti-inflammatory, and immunomodulatory properties, has emerged as a potential adjunctive therapeutic agent. The purpose of the present systematic review was to investigate whether systemic melatonin supplementation, when combined with NSPT, provides any additional periodontal and systemic benefits in patients diagnosed with type 2 diabetes mellitus (T2DM). **Methods**: Articles indexed in PubMed, Web of Science, Scopus, and the Cochrane Library were systematically retrieved, with additional screening of gray literature, covering all records available up to February 2026. This systematic review included randomized controlled trials (RCTs) evaluating the effect of adjunctive systemic melatonin administration in conjunction with NSPT on clinical and biochemical periodontal parameters and systemic outcomes related to glycemic control, oxidative stress, and inflammation in patients with T2DM compared with NSPT alone or combined with placebo. Three RCTs were eligible for qualitative synthesis, and two placebo-controlled RCTs were included in the quantitative synthesis. Risk of bias and quality of evidence assessments were performed. **Results**: With a low level of certainty, systemic melatonin supplementation in conjunction with NSPT demonstrated significantly more pronounced improvements in periodontal and systemic outcomes than those observed following NSPT or NSPT combined with placebo. Meta-analysis demonstrated statistically significant improvements in PPD and CAL, as well as in HbA1c and serum hs-CRP, favoring adjunctive melatonin supplementation. **Conclusions**: Adjunctive systemic melatonin supplementation in combination with NSPT may have a beneficial impact in patients with T2DM. However, the limited number of available studies, relatively small sample sizes, risk of bias concerns, and low quality of evidence limit the confidence that can be placed in these findings. Future research should focus on conducting more well-designed, sufficiently powered, large-scale RCTs employing standardized treatment protocols and longer observation periods.

## 1. Introduction

Periodontitis is a chronic multifactorial inflammatory disease defined by the progressive breakdown of the tooth-supporting tissues, driven by a complex and dynamic interplay between dysbiotic plaque biofilms and the host’s immune response [1]. Periodontitis and type 2 diabetes mellitus (T2DM) exhibit a well-established bidirectional relationship; evidence suggests that individuals with poorly controlled diabetes demonstrate an increased prevalence and severity of periodontitis, whereas periodontitis has been associated with impaired glycemic control and an elevated risk of diabetes-related complications [2,3,4,5,6,7,8,9]. This reciprocal link can be attributed to shared inflammatory, immunological, and metabolic mechanisms that contribute to the initiation and progression of both conditions [10,11,12].

In T2DM, chronic hyperglycemia promotes the accumulation of advanced glycation end products (AGEs) and enhanced AGE-RAGE binding, leading to elevated levels of oxidative stress and pro-inflammatory mediators, such as TNF-α, IL-1β, IL-6, and PGE_2_ [13,14,15,16,17,18]. The resulting persistent low-grade systemic inflammatory state, accompanied by impaired host defenses due to dysfunctional neutrophil and macrophage activity, facilitates periodontal inflammation [15,16,18]. In addition, collagen degradation, fibroblast dysfunction and diabetes-associated microangiopathy collectively impair periodontal tissue integrity and healing capacity, thereby contributing to periodontal destruction [19,20,21,22]. At the same time, periodontitis has also been consistently associated with impaired glycemic control in patients with diabetes [7,9,11]. Periodontal inflammation facilitates the systemic dissemination of pathogens, endotoxins, and pro-inflammatory mediators, thereby increasing systemic inflammatory burden and oxidative stress and promoting insulin resistance and impaired glycemic regulation [23,24,25,26,27,28,29,30,31,32,33]. However, these mechanisms have not yet been extensively investigated [34].

Notably, evidence indicates that non-surgical periodontal therapy (NSPT), primarily delivered through scaling and root planing (SRP), has been associated with favorable systemic effects and improved glycemic control in patients with T2DM, thereby further reinforcing the biological plausibility of the bidirectional link between the two diseases [35,36,37,38,39,40,41,42,43,44]. However, despite its established effectiveness in disrupting the microbial biofilm and reducing periodontal inflammation [45,46], numerous adjunctive strategies have been employed to overcome limitations in challenging cases and non-responsive sites, as well as in patients presenting with systemic conditions that can adversely affect periodontal healing, such as T2DM [47,48,49,50,51]. Local or systemic delivery of probiotics, antibiotics, and host-modulating agents can be used in conjunction with NSPT to enhance treatment outcomes; recent findings link these approaches with reduced inflammation, oxidative stress, and periodontal tissue breakdown [52,53,54,55,56,57,58,59,60].

Melatonin, a hormone that plays a substantial role in the regulation of circadian rhythms, has also been recognized for its antioxidant, anti-inflammatory, and immunomodulatory functions [61,62]. Within the oral environment, melatonin is detectable in saliva and gingival crevicular fluid (GCF) [63,64]. Decreased melatonin levels have been associated with periodontal inflammation [64,65,66,67]. Melatonin has been used adjunctively with NSPT via local delivery systems, such as orabase-based formulations [68,69,70,71], gels [72], and melatonin-loaded nanoparticles [73], as well as by systemic administration in the form of oral tablets [74]. The favorable outcomes that have been reported across studies [75,76] can be attributed to the diverse properties of melatonin, including the enhancement of antioxidant defense mechanisms, stimulation of collagen synthesis and angiogenic activity, promotion of osteoblast proliferation and differentiation, and suppression of osteoclast activity [77,78,79,80].

Emerging evidence indicates that melatonin is involved in metabolic regulation in individuals with diabetes mellitus [81]. The disease has been associated with suppressed melatonin secretion and reduced circulating levels of the hormone [82,83]. In this context, melatonin supplementation has been shown to have favorable outcomes on key metabolic and systemic parameters, including improvements in insulin sensitivity, reduction of systemic inflammation, and attenuation of oxidative stress [84,85]. Consequently, its adjunctive use in NSPT may be particularly beneficial in this population, as it may have a positive impact not only on periodontal inflammation but also on broader systemic benefits through the modulation of metabolic, inflammatory, and oxidative pathways implicated in the pathogenesis of both periodontitis and T2DM. Hence, the aim of this systematic review was to explore whether melatonin supplementation as an adjunct to NSPT provides additional periodontal and systemic benefits compared with NSPT alone or NSPT plus placebo in patients with T2DM.

## 2. Materials and Methods

### 2.1. Protocol and Registration

The present systematic review conformed to the Preferred Reporting Items for Systematic Reviews and Meta-Analyses (PRISMA) 2020 statement; in Supplementary Materials you can find the PRISMA checklist [86] (Supplementary File S1: PRISMA 2020 Checklist). Prospective registration of the review protocol was conducted before the initiation of this study. The corresponding protocol is available in the International Prospective Register of Systematic Reviews (PROSPERO) (registration number CRD420251155847).

### 2.2. Study Design

This systematic review addressed the following question: “Does systemic melatonin administration as an adjunct to NSPT improve clinical and biochemical periodontal parameters and the metabolic profile of patients with T2DM and periodontitis compared with NSPT or NSPT plus placebo?”

### 2.3. Eligibility Criteria

#### 2.3.1. Inclusion Criteria

Eligibility requirements were established following this study’s PICOS framework (Population, Intervention, Comparison, Outcomes, and Study design). Randomized controlled trials (RCTs) (S) evaluating the effect of systemically administered melatonin used adjunctively with NSPT (I) on periodontal parameters (O) in individuals diagnosed with T2DM (P), compared with the delivery of NSPT or NSPT combined with placebo (C), were considered eligible for inclusion in this systematic review. Further eligibility requirements comprised: clearly described treatment protocol regarding both NSPT and melatonin supplementation; adult human populations (≥18 years old); clinically established diagnosis of periodontitis; confirmed T2DM cases; and a minimum follow-up of 1.5 months. Only peer-reviewed articles written or translated into the English language were taken into consideration.

#### 2.3.2. Exclusion Criteria

Studies were not considered eligible if they involved study designs other than RCTs, such as non-randomized clinical trials, case reports and case series, literature reviews and systematic reviews, commentaries, editorials, personal opinions, book chapters, or conference abstracts, or if they evaluated local melatonin administration in the form of orabase-based creams, gels, or melatonin-loaded nanoparticles. Additional exclusion criteria comprised: studies enrolling patients with systemic comorbidities; studies including patients who had undergone periodontal treatment within the previous 6 months; studies involving smokers; studies including patients who had used antibiotics, anti-inflammatory, or antioxidant drugs within the preceding 6 months; studies enrolling pregnant or lactating women; studies without clearly defined treatment protocols; and in vitro experimental research or animal studies.

### 2.4. Information Sources and Search Strategy

The systematic search was completed through 5 February 2026. Tailored search strategies were designed by the two reviewers according to the syntax and indexing characteristics of the selected electronic databases (PubMed, Web of Science, Scopus, and Cochrane Library). A supplementary, targeted gray literature search was performed in Base, ProQuest, ResearchGate, and Google Scholar. Information regarding the detailed search strategy applied for each electronic database and gray literature source can be found in Table S1. Retrieved records were imported to Mendeley Reference Manager (version 2.144.0) for deduplication; any residual duplicate records were manually removed.

### 2.5. Study Selection

**Figure 1:**
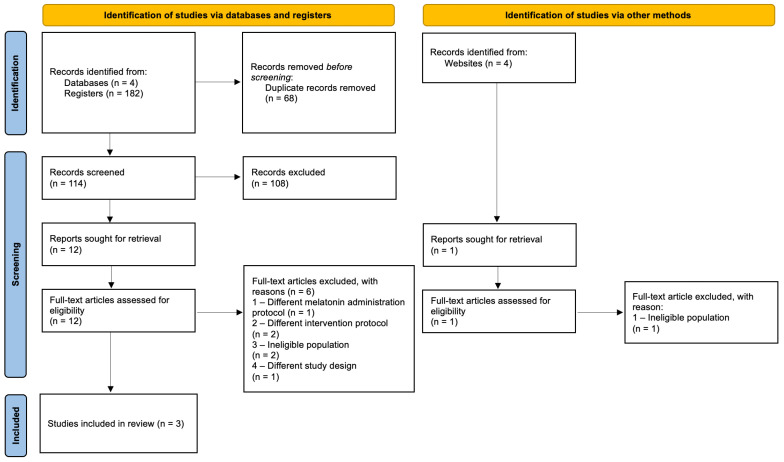
PRISMA flow diagram of the study selection process.

Both reviewers independently screened titles and abstracts and carried out full-text assessment of records selected for further evaluation. One author (T.A.) additionally screened all reference lists of these records to ensure no eligible reports were omitted. Any discrepancies arising between the two reviewers regarding article selection were addressed through discussion until consensus was achieved, while Cohen’s kappa coefficient [87] was calculated to assess the level of agreement between the two reviewers throughout this process. Figure 1 presents the PRISMA flow diagram, providing an overview of record retrieval, screening, eligibility assessment, and the final inclusion process.

### 2.6. Data Collection Process and Data Items

Data extraction from the included studies was performed independently by two reviewers, using a standardized Excel spreadsheet. For each eligible study, extracted variables included authorship, country of origin, publication year, journal, language, study design, sample size, participant demographic characteristics, and the diagnostic criteria used to define T2DM and periodontitis. The reviewers also collected and documented information regarding the NSPT protocol and the adjunctive melatonin regimen, including melatonin concentration, delivery route, dosing schedule, and treatment duration. Details about the length of follow-up periods and outcome measures assessed were also recorded.

### 2.7. Risk of Bias Within Studies

The included studies underwent independent risk of bias assessment by the two reviewers employing the Cochrane Risk of Bias tool for randomized trials (RoB 2) [88]. Evaluation across five domains resulted in an overall rating of “low risk of bias”, “some concerns”, or “high risk of bias”.

### 2.8. Summary Measures

The primary outcomes of this systematic review were changes reported in clinical periodontal parameters following periodontal treatment, whereas secondary outcomes included changes in biochemical periodontal parameters and changes in systemic parameters, including changes in parameters related to glycemic control, oxidative stress and systemic inflammation. More specifically, clinical periodontal outcomes included changes in probing pocket depth (PPD) and clinical attachment level (CAL), together with indices of gingival inflammation and plaque accumulation. Systemic effects were evaluated using measures of glycemic control and biomarkers of oxidative stress and inflammation.

### 2.9. Meta-Analysis

The mean changes and standard deviations from pre- and post-intervention values of examined parameters in the intervention and control groups were extracted. The effect size of systemic melatonin administration in the form of oral tablets in conjunction with NSPT on several periodontal indices and oxidative stress, inflammation, and glycemic control-related parameters in patients with T2DM was investigated using standardized mean difference (SMD; Cohen’s d) with 95% confidence interval (CI) using a random-effects model and RevMan 5.3 software (the Cochrane Collaboration, Copenhagen, Denmark). Data from a single follow-up time point (8 weeks post-treatment) were considered.

### 2.10. Risk of Bias Across Studies

The clinical heterogeneity of studies was considered by comparing variability in participants’ characteristics, interventions, and outcome measures. Methodological heterogeneity was assessed by evaluating differences in study design and risk of bias across included studies. Statistical heterogeneity across studies was evaluated using Cochran’s Q test and the I^2^ statistic, with I^2^ values interpreted as follows: low (<25%), moderate (25–50%), and high (>50%) heterogeneity, as well as through visual inspection of the forest plot. To further explore potential publication bias, funnel plot asymmetry was visually assessed, and Egger’s regression test was performed where appropriate.

### 2.11. Certainty of the Evidence

The overall confidence placed in the available evidence was determined using the GRADE framework. GRADE-pro software was used to generate the Summary of Findings (SoF) table (https://www.gradepro.org, accessed on 6 April 2026). The quality of evidence was classified as very low, low, moderate, or high, following risk of bias, inconsistency, indirectness, imprecision, and publication bias assessments, in addition to considerations regarding magnitude of effect, plausible residual confounding, and evidence of dose–response gradients.

## 3. Results

### 3.1. Study Selection

An initial search yielded 182 records from electronic databases, along with one additional record retrieved from gray literature sources. Following deduplication and preliminary title and abstract screening, 13 reports proceeded to full-text assessment, with seven of them being excluded at this stage because of differences in melatonin administration protocols (n = 1) [70], intervention protocol (n = 2) [89,90], population characteristics (n = 3) [91,92,93]), or study design (n = 1) [94] (Table S2). The level of inter-reviewer agreement was very high (Cohen’s κ > 0.90). No further eligible studies were retrieved through manual screening of reference lists. Therefore, three studies were included in the qualitative synthesis. The results of the study conducted by Bazyar et al. [95] were reported across three separate publications [95,96,97], and the results of Anton et al. [98] were presented in two different papers [98,99], each analyzing different outcomes within the same study population. The results of the third included study, conducted by Sarac Gul et al. [100], were published in a single paper, which could not, however, be included in the meta-analysis due to differences in the intervention protocol regarding melatonin supplementation, follow-up duration, and the lack of exact numerical data. Therefore, two studies [95,98] were pooled in a quantitative synthesis of periodontal and systemic outcomes, including PPD, CAL, HbA1c and serum hs-CRP, TNF-α, IL-6, and TAC.

### 3.2. Study Characteristics

All included studies were RCTs published in English during the period 2019–2024. The first study, by Bazyar et al. [95], was a double-blind, placebo-controlled RCT conducted in Iran. Its results were presented in three separate publications [95,96,97], each analyzing different outcomes within the same study population. This study included 44 participants equally allocated into two arms—the melatonin and placebo group—and a follow-up period of 8 weeks. Bazyar et al. [95] used the Eke et al. classification criteria [101] to define mild and moderate periodontitis cases and the ADA guidelines [102] to diagnose T2DM, while further excluding patients receiving insulin therapy or immunosuppressive medications. Participants in the test group received two 250 mg tablets of melatonin, each containing 3 mg of the antioxidant agent as an adjunct to NSPT, once daily, 1 h before bedtime for 8 consecutive weeks. The second study was also a double-blind, placebo-controlled RCT with 8-week follow-up, conducted by Anton et al. [98] in Romania, including 50 patients diagnosed with mild and moderate periodontitis and T2DM. The 25 participants allocated to the intervention group received two 250 mg tablets of melatonin, each containing 3 mg of the antioxidant agent, once daily, 1 h before bedtime for 8 consecutive weeks, whereas the control group received placebo tablets in conjunction with NSPT. Diagnosis was based on the classification of the Eke et al. criteria [101] and the ADA guidelines [102], respectively. Anton et al. [98] also further excluded patients receiving insulin therapy or immunosuppressive medications. The results of this trial were published in two different papers [98,99]. The last included study was conducted by Sarac Gul et al. [100] in Turkey, and it employed a single-blind RCT design, recruiting 55 participants diagnosed with Stage III or IV grade C periodontitis and T2DM. Periodontitis was diagnosed according to the Tonetti et al. [103] classification system, and T2DM was diagnosed using the ADA guidelines [102], hence all participants met the diagnostic thresholds of FBS ≥ 126 mg/dL and HbA1c ≥ 6.5%, or 2 h glucose (2-hpp) ≥ 200 mg/dL. Sarac Gul et al. [100] additionally excluded night shift workers from the population of their study. The melatonin-treated group in this trial received 250 mg tablets of melatonin, each containing 3 mg of the antioxidant agent as an adjunct to NSPT, once daily, 1 h before bedtime for 30 days, while the control group underwent NSPT alone. This study included a 3- and 6-month follow-up period. Overall, a total of 149 subjects participated in the included studies. Regarding sample size planning, all three included RCTs reported an a priori estimation of the required sample size. Bazyar et al. [95] calculated the sample size based on IL-6 as the main variable and recruited 25 participants per group after accounting for possible withdrawals. Anton et al. [98] performed a power analysis based on an expected between-group difference in HbA1c and similarly increased the calculated sample size to account for potential dropouts. Sarac Gul et al. [100] used G*Power software, with PPD as the primary outcome, and determined that at least 52 participants were required. Therefore, all included trials provided evidence of prospective sample size planning, although the final sample sizes remained relatively small. Across trials, NSPT comprised scaling and root planing (SRP) combined with oral hygiene instructions (OHI). Patients with systemic comorbidities; those who had used antibiotics, anti-inflammatory or antioxidant drugs within the preceding 6 months; patients who had recently received periodontal therapy; smokers; and pregnant or lactating women were excluded from all studies. Follow-up periods ranged from 2 [95,98] to 6 months [100]. All included RCTs reported ethical approval from the respective institutional ethics committees and obtained informed consent from participants. An overview of the descriptive characteristics of the included studies is provided in Table 1.

### 3.3. Risk of Bias Within Studies

**Figure 2:**
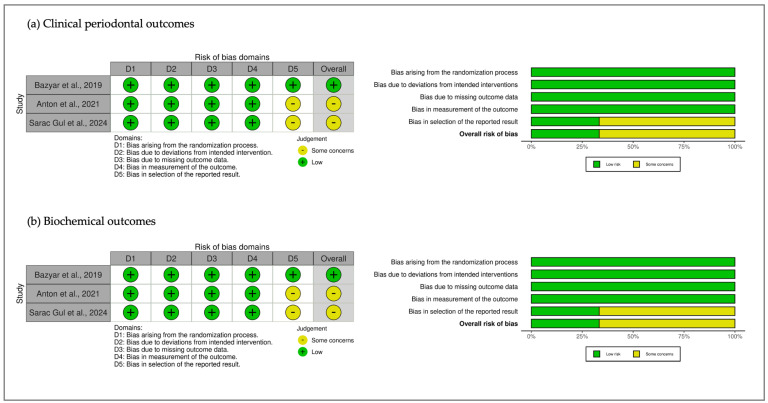
Risk of bias evaluation of the RCTs included in the review. Risk of bias was appraised across five domains with the use of the RoB 2 tool (Version 2019) [88] and graphically displayed using the robvis tool [104]. (**a**) Clinical periodontal outcomes: the (**left**) panel shows the study-specific judgments for each bias domain, whereas the (**right**) panel summarizes the distribution of domain-level judgments across the included studies [95,98,100]. (**b**) Biochemical outcomes: the (**left**) panel shows the study-specific judgments for each bias domain, whereas the (**right**) panel summarizes the distribution of domain-level judgments across the included studies [95,98,100].

An overview of the risk of bias assessments for the three included RCTs is presented in Figure 2. One study [95] received a “low risk” of bias judgment across all evaluated domains. The two remaining trials [98,100] were rated as presenting “some concerns”, primarily attributable to inadequate reporting of preregistered study protocols.

### 3.4. Synthesis of Results and Assessment of the Certainty of Evidence

#### 3.4.1. Changes in Periodontal Outcomes

All included trials assessed the effect of systemic melatonin supplementation in conjunction with NSPT on both clinical and biochemical periodontal parameters. Clinical periodontal outcomes, including changes in PPD, CAL, PI, and indices reflecting gingival inflammation, such as BoP and GI, were evaluated in all three included studies. Overall, results from both the test and control groups consistently demonstrated improvements in clinical periodontal parameters following NSPT, both with and without adjunctive melatonin supplementation, with melatonin-treated groups generally exhibiting greater improvements. Nevertheless, not all trials consistently demonstrated statistically significant intergroup differences.

In the study of Bazyar et al. [95], recruiting 44 participants equally distributed between the control and test groups, the mean CAL significantly decreased in both groups 8 weeks after periodontal therapy. Mean PPD values were significantly reduced only in the test group. Overall, the melatonin-treated group showed statistically greater improvement than the placebo-controlled group (*p* < 0.001). BoP and PI showed no significant changes following treatment. More specifically, in the test group, mean PPD decreased from 4.45 ± 0.96 mm at baseline to 2.59 ± 1.04 mm post-treatment (*p* < 0.001), and mean CAL showed a similar reduction, decreasing from 3.04 ± 0.78 mm at baseline to 1.59 ± 0.59 mm (*p* < 0.001), while in the placebo group, mean PPD decreased from 4.54 ± 1.01 mm at baseline to 4.36 ± 1.04 mm post-treatment without reaching statistical significance, and mean CAL decreased from 3.00 ± 0.75 mm at baseline to 2.77 ± 0.68 mm (*p* = 0.021), respectively.

Results from the RCT conducted by Anton et al. [98] indicated that melatonin administration had a beneficial impact on periodontal indices. The study included 50 participants, out of whom 25 were assigned to the placebo control group. Mean values of PPD and CAL decreased significantly only in the melatonin group (*p* < 0.001), whereas BoP and PI showed statistically significant reductions in both groups (*p* < 0.001, test group; *p* < 0.05, control group). Significant intergroup differences were observed for PPD and CAL (*p* < 0.001) but not for BoP nor PI. More specifically, Anton et al. reported that mean PPD and CAL in the melatonin-treated group decreased from 4.65 ± 1.04 mm and 3.05 ± 0.56 mm at baseline to 2.27 ± 0.7 mm and 1.24 ± 0.45 mm 8 weeks post-treatment, respectively (*p* < 0.001), and from 4.53 ± 1.01 mm and 3.02 ± 0.93 mm at baseline to 4.40 ± 1.02 mm and 2.98 ± 0.96 mm following periodontal therapy in the placebo group without reaching statistical significance.

Sarac Gul et al. [100], in an RCT comprising 55 participants, reported significant improvements in clinical periodontal parameters in both groups at 3 and 6 months post-treatment (*p* < 0.05). Intergroup comparisons showed that melatonin supplementation led to greater reductions in PPD, BoP, and GI at 3 months and in PI and BoP at 6 months, compared to the group receiving NSPT alone (*p* < 0.05). In the same study, GCF MMP-8, RANKL, and OPG concentrations were also assessed. GCF MMP-8 and GCF RANKL levels decreased at both 3- and 6-month follow-ups in both groups (*p* < 0.05), whereas GCF OPG levels were significantly lower only at 6 months and only in the control group (*p* < 0.05). Due to this limited change in OPG, the RANKL/OPG ratio remained similar between groups. Intergroup comparisons revealed that melatonin administration was associated with a greater reduction in MMP-8 and RANKL levels at 3 months.

**Figure 3:**
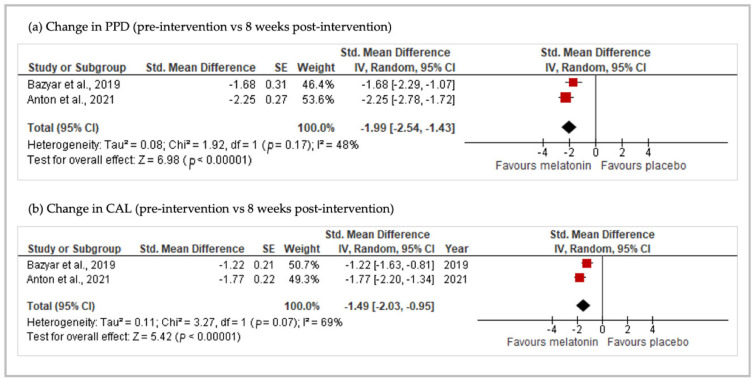
Forest plots showing standardized mean difference in (**a**) PPD, as compared between pre-and post-intervention, and (**b**) CAL, as compared between pre- and post-intervention for the studies pooled in the meta-analysis [95,98].

Overall, meta-analysis findings showed that melatonin supplementation as an adjunct to NSPT significantly reduced PPD (SMD = −1.99, 95% CI: −2.54 to −1.43, *p* < 0.00001), indicating a moderate to large effect of melatonin supplementation, with moderate heterogeneity (I^2^ = 48%). Similarly, CAL was significantly improved (SMD = −1.49, 95% CI: −2.03 to −0.95, *p* < 0.00001), although substantial heterogeneity was observed (I^2^ = 69%), suggesting variability in treatment effects across studies (Figure 3). The quality of evidence for these outcomes was judged to be low (Table S3).

#### 3.4.2. Changes in Systemic Outcomes

##### Changes in Glycemic Control

Bazyar et al. [95] reported significant reductions in mean serum levels of HbA1c in the melatonin-treated group, both within-group (8.64% ± 1.66% at baseline vs. 7.67% ± 1.19% at 8 weeks post-treatment, *p* = 0.004) and in comparison with the control group (mean change: test group: −0.97% ± 1.42% vs. control group: −0.18% ± 0.72%, *p* = 0.02), with these differences remaining significant after adjustment for confounding factors. Serum FBG levels showed a nonsignificant decrease in the melatonin group. Anton et al. [98] also reported significant improvements in serum HbA1c levels in the intervention group receiving melatonin supplementation (7.6243% ± 0.71% at baseline vs. 6.2781% ± 0.31% at 8 weeks post-treatment, *p* < 0.001), with greater improvements reported compared to the placebo group (*p* < 0.001).

**Figure 4:**
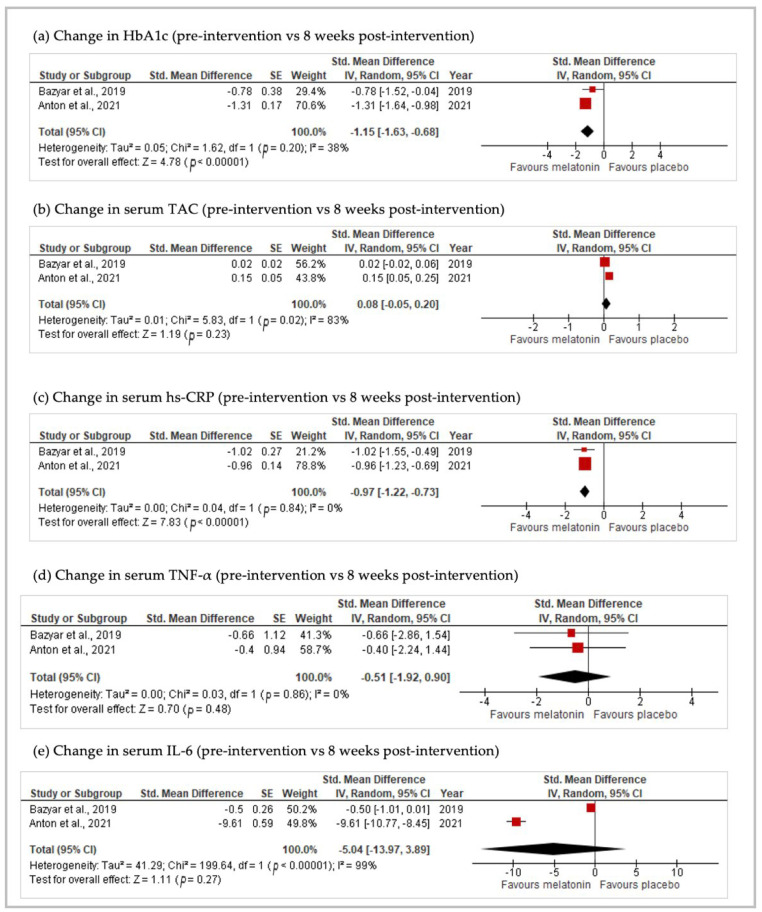
Forest plots showing standardized mean difference in (**a**) HbA1c, as compared between pre-and post-intervention, (**b**) serum TAC, as compared between pre- and post-intervention, (**c**) hs-CRP, as compared between pre- and post-intervention, (**d**) serum TNF-α, as compared between pre- and post-intervention, and (**e**) serum IL-6, as compared between pre- and post-intervention for the studies pooled in the meta-analysis [95,98].

Meta-analysis findings showed that melatonin supplementation as an adjunct to NSPT led to a statistically significant improvement in HbA1c (SMD = −1.15 (95% CI: −1.63 to −0.68, *p* < 0.00001), indicating a moderate effect favoring melatonin supplementation, with moderate heterogeneity (I^2^ = 38%) (Figure 4a). The certainty of the available evidence regarding these outcomes was considered to be low (Table S3).

##### Changes in Oxidative Stress Parameters

Bazyar et al., in one of their subsequent publications [96], found a significant reduction in the mean serum MDA levels only in the melatonin-treated group (17.2 ± 1.82 μΜ at baseline to 16.13 ± 1.76 μΜ post-treatment, *p* < 0.001). The reduction in MDA levels in the test group was significantly greater than in the control group (*p* = 0.008) and remained significant after adjustment for confounding factors. With regard to SOD, CAT, TAC, and GPx, significant increases were observed in the mean serum levels of these oxidative stress biomarkers in the melatonin group compared with the control group (SOD levels increased from 13.91 ± 2.75 U/mL at baseline to 15.53 ± 4.37 U/mL 8 weeks post-treatment, *p* = 0.008; GPx levels from 243.04 ± 68.37 U/mL to 262.04 ± 62.45 U/mL post-treatment, *p* = 0.004; CAT levels from 24.23 ± 4.54 U/mL to 27.47 ± 4.12 U/mL, *p* = 0.004; TAC levels from 0.289 ± 0.04 mM at baseline to 0.313 ± 0.05 mM 8 weeks post-treatment, *p* = 0.02). Apart from TAC, the magnitude of these changes was significantly greater in the intervention group compared with the control group (*p* = 0.02, *p* = 0.04, and *p* = 0.04, respectively). These findings remained significant after adjustment for confounding factors.

Results from Anton et al. [98] demonstrated significant increases in saliva and serum TAC and reductions in TOS levels only in the intervention group at 8 weeks post-treatment. More specifically, saliva and serum TAC levels decreased from 0.57 ± 0.11 μmol and 1.27 ± 0.21 μmol at baseline to 1.32 ± 0.26 μmol and 1.41 ± 0.24 μmol post-treatment, respectively (*p* < 0.001), while saliva and serum TOS levels decreased from 0.073 ± 0.009 μmol and 0.016 ± 0.004 μmol at baseline to 0.031 ± 0.001 μmol and 0.01 ± 0.004 μmol post-treatment, respectively (*p* < 0.05). Overall, these effects were more pronounced in the melatonin-treated group (*p* < 0.001).

Meta-analysis findings showed no statistically significant effect of melatonin on serum TAC (SMD = 0.08, 95% CI: −0.05 to 0.20, *p* = 0.23), with considerable heterogeneity (I^2^ = 83%), indicating inconsistent findings across studies (Figure 4b). The level of certainty in the evidence for these outcomes was judged to be low (Table S3).

##### Changes in Inflammatory Parameters

Bazyar et al. [95] found significantly decreased mean serum levels of IL-6 and hs-CRP 8 weeks post-treatment only in the melatonin-treated group (IL-6 decreased from 2 ± 0.92 at baseline to 1.42 ± 0.73 pg/mL post-treatment, *p* = 0.008; hs-CRP decreased from 2.53 ± 0.77 μg/L to 1.6 ± 0.91 mg/L, *p* = 0.017). Notably, mean changes in the serum IL-6 and hs-CRP levels were significantly (*p* = 0.04, *p* = 0.007) lower in the intervention group compared to the control group. In one of this study’s [95] subsequent publications, Javid et al. [96] reported a significant decrease in the mean serum IL-1β levels only in the melatonin-treated group (2.41 ± 0.55 pg/mL at baseline to 2.06 ± 0.48 pg/mL post-treatment, *p* = 0.008). No statistically significant differences were observed between groups 8 weeks following NSPT.

In the study of Anton et al. [98], reductions in saliva and serum IL-6 and hs-CRP concentrations were observed after treatment. However, these changes reached statistical significance only in the melatonin-treated group (saliva and serum IL-6 decreased from 2.09 ± 0.60 ng/mL and 20.54 ± 2.0 ng/mL at baseline to 1.32 ± 0.32 ng/mL and 10.11 ± 1.6 ng/mL post-treatment, respectively, *p* < 0.001; saliva and serum hs-CRP decreased from 2.73 ± 0.71 ng/mL and 2.24 ± 0.51 ng/mL at baseline to 1.24 ± 0.46 ng/mL and 1.14 ± 0.62 ng/mL post-treatment, respectively, *p* < 0.001). The melatonin-treated group showed more pronounced improvements for both outcomes (*p* < 0.001). Saliva and serum TNF-α levels showed nonsignificant reductions.

Sarac Gul et al. [100] also reported that serum IL-1β levels were significantly lower in both groups at 3 and 6 months post-treatment (*p* < 0.05). Intergroup analysis showed a greater reduction in serum IL-1β levels in the melatonin group at 6 months compared to the control group (*p* < 0.05).

Overall, the meta-analysis demonstrated that melatonin supplementation as an adjunct to NSPT resulted in a statistically significant reduction in hs-CRP levels (SMD = −0.97 (95% CI: −1.22 to −0.73, *p* < 0.00001), indicating a moderate and consistent effect favoring melatonin supplementation, with no observed heterogeneity (I^2^ = 0%), indicating consistent effects across studies (Figure 4c). In contrast, no significant effect was observed for serum TNF-α levels, showing a non-significant reduction (SMD = −0.51, 95% CI: −1.92 to 0.90, *p* = 0.48) and no heterogeneity (I^2^ = 0%) (Figure 4d). Similarly, serum IL-6 levels did not show a statistically significant change (SMD = −5.04 (95% CI: −13.97 to 3.89, *p* = 0.27), with considerable heterogeneity (I^2^ = 99%) (Figure 4e). The overall quality of evidence for these outcomes was rated as low (Table S3).

## 4. Discussion

### 4.1. Summary of Evidence

The present systematic review evaluated whether systemic melatonin supplementation provides additional periodontal and systemic benefits when used as an adjunct to NSPT in patients with T2DM and periodontitis. Three RCTs [95,98,100], comprising a total of 149 participants, were included in the qualitative synthesis, whereas two placebo-controlled randomized trials [95,98] with 94 participants were eligible for quantitative synthesis. Overall, adjunctive systemic melatonin administration, in conjunction with NSPT, was associated with more favorable outcomes than control interventions, which could be either NSPT alone or NSPT plus placebo. Although both groups showed generally improved outcomes following NSPT, statistically significant and more pronounced improvements were consistently observed in the melatonin-treated groups, whereas the control groups often showed smaller and, in several cases, non-significant changes.

The findings of this systematic review suggest that adjunctive melatonin supplementation was associated with improved periodontal healing following NSPT. Both pooled RCTs conducted by Bazyar et al. [95] and Anton et al. [98] demonstrated significant reductions in PPD and CAL favoring melatonin, and these findings were further supported by the additional study of Sarac Gul et al. [100], which reported greater reductions in PPD at 3 months and sustained improvements in other clinical indices in a follow-up of 6 months. Although periodontal indices related to inflammation, such as BoP, GI, and PI, were not quantitatively pooled, narrative synthesis reveals a tendency toward improved infection control in the melatonin-treated groups. In addition to these clinical findings, the study of Sarac Gul et al. [100] indicated favorable effects on biochemical periodontal outcomes related to connective tissue degradation and bone metabolism, particularly through greater reductions in GCF MMP-8 and RANKL levels, whereas no clear effect was observed for GCF OPG levels or the RANKL/OPG ratio. The biological plausibility of the favorable outcomes following melatonin administration can be attributed to the antioxidant, anti-inflammatory, and immunomodulatory properties of melatonin and its beneficial effect on collagen synthesis, angiogenesis, osteoblastic activity, and wound healing, all of which may support periodontal healing [61,62,77,78,79,80].

As for systemic outcomes, and more specifically glycemic control, pooled analysis demonstrated a statistically significant reduction in HbA1c, indicating that the adjunctive use of systemic melatonin may favorably influence metabolic control in patients with T2DM undergoing periodontal treatment. Although serum FBG did not improve significantly in the included studies, the consistent HbA1c reduction is in agreement with the reported beneficial effects of melatonin on glycemic regulation in this population and with the growing interest in melatonin supplementation as a strategy to improve metabolic control in patients with T2DM [81,105,106].

Regarding inflammatory outcomes, pooled evidence revealed a statistically significant reduction in serum hs-CRP, indicating that adjunctive melatonin supplementation may enhance systemic inflammation control beyond the effects of NSPT alone. Although pooled effects for serum TNF-α and IL-6 were not significant, the overall direction of evidence, further supported by reductions in serum IL-1β in non-pooled analyses, remained favorable toward melatonin, suggesting a potential adjunctive anti-inflammatory effect. These findings align with the existing literature suggesting that NSPT may contribute to the reduction of systemic inflammatory burden in patients with T2DM [35,36,37,38,39,40,41,42,43,44], while also reinforcing the view that adjunctive melatonin supplementation may provide additional anti-inflammatory benefits beyond those achieved with NSPT alone [40,107,108,109].

Findings associated with oxidative stress were also heterogeneous. Pooled analysis of serum TAC did not demonstrate a statistically significant effect and additionally showed considerable between-study heterogeneity. However, individual trial data suggested significant improvements in several oxidative stress biomarkers, including reductions in MDA and TOS and increases in SOD, CAT, and GPx in the melatonin-treated groups. Hence, melatonin administration combined with NSPT had a beneficial therapeutic effect on the overall oxidative stress profile of these patients compared with NSPT plus placebo, therefore reinforcing melatonin’s well-established antioxidant properties and capacity to scavenge free radicals, and overall enhancing the antioxidant defense systems [62,84,85,110,111,112].

These results are also compatible with available evidence investigating the effect of melatonin administration as an adjunct to NSPT using local delivery systems, as favorable periodontal and anti-inflammatory effects have been reported when using orabase-based formulations [68,69,70,71], gels [72], and melatonin-loaded nanoparticles [73] in conjunction with NSPT. Likewise, following systemic melatonin supplementation, previous trials have demonstrated beneficial effects of its administration in the form of oral tablets in both systemically healthy individuals and patients with diabetes, suggesting that melatonin may improve periodontal parameters and reduce inflammatory burden [74,75,76]. Therefore, melatonin may represent a promising adjunctive host-modulating strategy in periodontitis management, especially in patients with T2DM or other systemic diseases characterized by low-grade inflammation and impaired healing capacity.

The statistically significant improvements observed in PPD, CAL, HbA1c, and hs-CRP may have clinical relevance; however, this interpretation should be made cautiously. The greater reductions in PPD and CAL observed in the melatonin-treated groups suggest that systemic melatonin may enhance the local periodontal response to NSPT, particularly in patients with T2DM, in whom impaired inflammatory regulation, oxidative stress, and compromised healing may limit treatment responsiveness [113,114]. In this context, even modest additional improvements in periodontal parameters may be meaningful, as residual pocket depth and attachment loss are clinically relevant indicators of periodontal stability and future treatment needs. Regarding HbA1c, the observed reduction may be clinically relevant in the context of periodontal therapy in patients with T2DM. NSPT has been associated with modest improvements in glycemic control, although the magnitude of this effect is generally limited and variable across studies [35,37,39,42,43,44]. Therefore, the additional reduction in HbA1c observed with adjunctive melatonin may indicate a potential additive benefit beyond NSPT alone. However, given the small number of eligible studies, the relatively small sample sizes, short follow-up periods, and low certainty of evidence, it remains unclear whether these differences are clinically meaningful, and the findings should be interpreted with caution.

### 4.2. Limitations and Strengths

Despite the high biological plausibility supporting the association between melatonin administration as an adjunct to NSPT and favorable periodontal and systemic outcomes, the findings of this systematic review require careful interpretation. The main limitation of this systematic review is the small amount of available evidence. After a comprehensive systematic search of the existing literature, only three RCTs met the eligibility criteria and were included in this review, and only two of them could be quantitatively synthesized. This resulted in a relatively small cumulative sample size and reduced the precision of pooled estimates. Furthermore, the meta-analysis of HbA1c was based on change-from-baseline values, as consistently reported baseline-adjusted estimates were not available across the included trials. In addition to that, heterogeneity between studies arose from differences in study populations and methodological characteristics. Although all included participants were diagnosed with T2DM and periodontitis, Bazyar et al. [95] and Anton et al. [98] recruited patients with mild to moderate periodontitis diagnosed according to Eke et al. classification criteria [101], whereas Sarac Gul et al. [100] enrolled patients with stage II or IV grade C periodontitis according to the 2018 classification [103]. These differences in periodontal case definition and severity could potentially affect treatment responsiveness. Furthermore, follow-up duration ranged from 8 weeks in the pooled trials [95,98] to 3 and 6 months in the third study [100], thereby limiting comparability. Methodological heterogeneity was also evident in intervention protocols and outcome reporting. Notably, the control groups in two of the included trials consisted of patients receiving placebo tablets as a supplement to NSPT [95,98], whereas in one study, the control group received NSPT alone [100] and not all trials were designed to explore the same scope of periodontal and systemic outcomes. Moreover, two of the included studies [98,100] were judged as raising some concerns in terms of risk of bias, primarily due to inadequate information on preregistered study protocols, whereas only one study [95] was assessed as being at low risk of bias. Accordingly, the overall quality of evidence across the outcomes was low. Hence, the findings of this systematic review should be interpreted cautiously and regarded as exploratory rather than definitive and confirmatory. Given the limited certainty of the available evidence, firm conclusions cannot be supported, while the interpretation and broader applicability of the review findings remain restricted.

Despite these limitations, this systematic review also presents several important strengths. The review question was clearly structured according to the PICOS framework, the protocol was prospectively registered in PROSPERO, and the study was conducted according to PRISMA 2020 recommendations [86]. Four major scientific databases, along with the gray literature, were comprehensively searched. Only RCTs were included so that methodological consistency and reliable comparisons of treatment effects would be ensured. Study screening, data collection, and risk of bias evaluation were undertaken independently by two reviewers. Discrepancies between reviewers were discussed until consensus was achieved. Moreover, well-established and validated Cochrane methodological tools were employed to appraise both the risk of bias and the overall certainty of the evidence within the included studies. An additional strength is that the included studies were all RCTs and provided data on both periodontal and systemic outcomes, allowing a more thorough assessment of melatonin’s potential effects in T2DM patients undergoing periodontal treatment and a comprehensive synthesis of the available data, while also highlighting important limitations.

## 5. Conclusions

Systemic melatonin supplementation as an adjunct to NSPT appears to provide additional benefits in patients with T2DM and periodontitis, particularly with respect to clinical periodontal improvement, glycemic control, and selected systemic inflammatory and oxidative stress-related outcomes. However, considering the currently limited body of evidence, further well-designed and sufficiently powered RCTs employing standardized treatment protocols and integrating longer observation periods are required to validate these findings.

## Figures and Tables

**Table 1:** Summary of descriptive characteristics of included studies.

Study/ Country	Study Design	Study Population, Intervention and Control Groups, n	Age (Years)	Periodontitis and Diabetes Definition	Intervention Protocol	Periodontal and Systemic Outcomes	Follow-Up (Months)	Main Conclusion
Bazyar et al., 2019 [95] Javid et al., 2020 [96], Bazyar et al., 2022 [97] Iran	Double-blind, placebo-controlled RCT	44 participants: melatonin group, 22 control group, 22	53.72 ± 6.68 (melatonin group) 51.45 ± 5.03 (control group)	Mild and moderate periodontitis (PPD ≥ 4 mm and CAL = 1–4 mm) [101] FBS ≥ 126 mg/dL and HbA1c ≥ 6.5% or 2 h glucose (2-hpp) ≥ 200 mg/dL [102]	NSPT (OHI, SRP) + Melatonin tablets (6 mg/day/8 weeks) (melatonin group) NSPT (OHI, SRP) + Placebo tablets (control group)	PPD, CAL, BoP, PI Serum TNF-α, IL-6, hs-CRP [95] Serum IL-1β, MDA, TAC, SOD, CAT, GPx [96] FBG, HbA1c [97]	2	Systemic melatonin supplementation as an adjunct to NSPT significantly improved periodontal parameters and reduced inflammation and oxidative stress, while also improving antioxidant capacity and glycemic control in patients with T2DM.
Anton et al., 2021 [98] Anton et al., 2022 [99] Romania	Double-blind, placebo-controlled RCT	50 participants: melatonin group, 25 control group, 25	53.24 ± 3.4 (melatonin group) 52.21 ± 3.1 (control group)	Mild and moderate periodontitis (PPD ≥ 4 mm and CAL = 1–4 mm) [101] FBS ≥ 126 mg/dL and HbA1c ≥ 6.5% or 2 h glucose (2-hpp) ≥ 200 mg/dL [102]	NSPT (OHI, SRP) + Melatonin tablets (6 mg/day/8 weeks) (melatonin group) NSPT (OHI, SRP) + Placebo tablets (control group)	PPD, CAL, BoP, PI, HbA1c [98] Saliva and serum hs-CRP, TNF-α, IL-6, TAC, TOS [99]	2	Systemic melatonin supplementation in conjunction with NSPT significantly improved periodontal parameters and glycemic control, while also significantly reducing inflammatory and oxidative stress markers and increasing antioxidant capacity in patients with T2DM.
Sarac Gul et al., 2024 [100] Turkey	Single-blind RCT	55 participants: melatonin group, 28 control group, 27	51.85 ± 8.58 (melatonin group) 54.07 ± 6.22 (control group)	Stage III or IV, grade C periodontitis [103] FBS ≥ 126 mg/dL and HbA1c ≥ 6.5% or 2 h glucose (2-hpp) ≥ 200 mg/dL [102]	NSPT (OHI, SRP) + Melatonin tablets (6 mg/day/30 days) (melatonin group) NSPT (OHI, SRP) (control group)	PPD, CAL, BoP, GI, PI GCF MMP-8, GCF RANKL, GCF OPG, RANKL/OPG ratio Serum IL-1β	3, 6	Systemic melatonin supplementation in conjunction with NSPT significantly improved periodontal parameters, while also significantly reducing biochemical markers related to inflammation, connective tissue breakdown, and bone resorption in patients with T2DM.

RCT, randomized controlled trial; FBS, fasting blood sugar; HbA1c, hemoglobin A1c; 2-hpp, 2 h postprandial plasma (glucose); PPD, probing pocket depth; CAL, clinical attachment level; PI, plaque index; BoP, bleeding on probing; *GI*, gingival index; hs-CRP, high-sensitivity C-reactive protein; MDA, malondialdehyde; TAC, total antioxidant capacity; SOD, superoxide dismutase; CAT, catalase; GPx, glutathione peroxidase; GCF, gingival crevicular fluid; IL-1β, Interleukin-1-beta; IL-6, interleukin-6; TNF-α, tumor necrosis factor alpha; MMP-8, matrix metalloproteinase-8; RANKL, Receptor activator of nuclear factor kappa-b; OPG, osteoprotegerin, NSPT, non-surgical periodontal therapy; SRP, scaling and root planing; OHI, oral hygiene instructions.

## Data Availability

No new data were created or analyzed in this study. Data sharing is not applicable to this article.

## Supplementary Materials

The following supporting information can be downloaded at: https://www.mdpi.com/article/10.3390/jcm15114071/s1, Table S1: Detailed search strategy for electronic databases; Table S2: Excluded articles and reasons for exclusion; Table S3: Quality assessment of included studies. File S1: PRISMA 2020 Checklist.

## Abbreviations

The following abbreviations are used in this manuscript:

NSPT	Non-surgical periodontal therapy
T2DM	Type 2 diabetes mellitus
RCT	Randomized controlled trial
AGEs	Advanced glycation end products
PGE2	Prostaglandin E2
FBS	Fasting blood sugar
HbA1c	Hemoglobin A1c
2-hpp	2 h postprandial plasma (glucose)
PPD	Probing pocket depth
CAL	Clinical attachment level
BoP	Bleeding on probing
PI	Plaque index
GI	Gingival index
hs-CRP	High-sensitivity C-reactive protein
MDA	Malondialdehyde
TAC	Total antioxidant capacity
SOD	Superoxide dismutase
CAT	Catalase
GPx	Glutathione peroxidase
GCF	Gingival crevicular fluid
IL-1β	Interleukin-1-beta
IL-6	Interleukin-6
TNF-α	Tumor Necrosis Factor alpha
MMP-8	Matrix metalloproteinase-8
RANKL	Receptor activator of nuclear factor kappa-b
OPG	Osteoprotegerin
OHI	Oral hygiene instructions
SRP	Scaling and root planing
