# Supplementary material for: Effect of Melatonin as an Adjunct to NSPT on Periodontal and Systemic Outcomes in Patients with Type 2 Diabetes Mellitus: A Systematic Review and Meta-Analysis of RCTs
Source: J Clin Med. 2026 May 25;15(11):4071. doi: 10.3390/jcm15114071 (PMC13257706; doi:10.3390/jcm15114071)
Supplement: Supplementary file 1 [file jcm-15-04071-s001.zip › jcm-4294332-supplementary/Supplementary Files_Effect of Melatonin as an Adjunct to NSPT on Periodontal and Systemic Outcomes in Patients with Type 2 Diabetes Mellitus A Systematic Review and Meta-Analysis of RCTs.pdf]

Systematic Review

Effect of Melatonin as an Adjunct to NSPT on Periodontal and Systemic Outcomes in Patients with Type 2 Diabetes Mellitus: A Systematic Review and Meta-Analysis of RCTs

Thaleia Angelopoulou [1] and Yiorgos A. Bobetsis[2,*]

[1] DDS, Postgraduate Student, School of Medicine, National and Kapodistrian University of Athens, 11527 Athens, Greece; thangelop@yahoo.gr

[2] Associate Professor, Department of Periodontology, School of Dentistry, National and Kapodistrian University of Athens, 11527 Athens, Greece; ybobetsi@dent.uoa.gr

* Correspondence: ybobetsi@dent.uoa.gr; Tel.: +306936613292

Supplementary Material

Table S1. Detailed search strategy for electronic databases.

Electronic databases

Database	Search (November 5, 2026)	Studies
**PubMed**	("Periodontitis"[MeSH] OR "Periodontal Diseases"[MeSH] OR periodontitis[tiab] OR periodontal[tiab] OR "Dental Scaling"[MeSH] OR "Debridement"[MeSH] OR "non-surgical periodontal therapy"[tiab] OR "periodontal therapy"[tiab] OR "periodontal treatment"[tiab] OR "scaling and root planing"[tiab] OR NSPT[tiab] OR SRP[tiab] OR PDT[tiab]) AND ("Diabetes Mellitus, Type 2"[MeSH] OR "type 2 diabetes"[tiab] OR "type 2 diabetes mellitus"[tiab] OR T2DM[tiab] OR diabetes[tiab] OR diabetic[tiab]) AND ("Melatonin"[Mesh] OR melatonin[tiab] OR "systemic melatonin"[tiab] OR "oral melatonin"[tiab] OR "melatonin supplementation"[tiab] OR "melatonin therapy"[tiab] OR "melatonin administration"[tiab] OR "exogenous	23

	melatonin"[tiab] OR "melatonin intake"[tiab] OR "melatonin treatment"[tiab] OR "melatonin use"[tiab] OR "melatonin tablets"[tiab] OR "melatonin pills"[tiab])	
**Web of Science**	(Periodontitis OR periodontal disease OR periodontal therapy OR dental scaling OR debridement OR non-surgical periodontal therapy OR periodontal treatment OR scaling and root planing OR NSPT OR SRP OR PDT) AND (type 2 diabetes OR type 2 diabetes mellitus OR T2DM OR diabetes OR diabetic) AND (Melatonin OR systemic melatonin OR oral melatonin OR melatonin supplementation OR melatonin therapy OR melatonin administration OR exogenous melatonin OR melatonin intake OR melatonin treatment OR melatonin use OR melatonin tablets OR melatonin pills)	94
**Scopus**	TITLE-ABS-KEY ("Periodontitis" OR "Periodontal Diseases" OR "Dental Scaling" OR "Debridement" OR "non-surgical periodontal therapy" OR "periodontal therapy" OR "periodontal treatment" OR "scaling and root planing" OR NSPT OR SRP OR PDT) AND ("type 2 diabetes" OR "type 2 diabetes mellitus" OR T2DM OR diabetes OR diabetic) AND ("Melatonin" OR melatonin OR "systemic melatonin" OR "oral melatonin" OR "melatonin supplementation" OR "melatonin therapy" OR "melatonin administration" OR "exogenous melatonin" OR "melatonin intake" OR "melatonin treatment" OR "melatonin use" OR "melatonin tablets" OR "melatonin pills")	50
**Cochrane Library**	(Periodontitis OR periodontal disease OR periodontal therapy OR dental scaling OR debridement OR non-surgical periodontal therapy OR periodontal treatment OR scaling and root planing OR NSPT OR SRP OR PDT) AND (type 2 diabetes OR type 2 diabetes mellitus OR T2DM OR diabetes OR diabetic) AND (Melatonin OR systemic melatonin OR oral melatonin OR melatonin supplementation OR melatonin therapy OR melatonin administration OR exogenous melatonin OR melatonin intake OR melatonin treatment OR melatonin use OR melatonin tablets OR melatonin pills)	15

Grey Literature

**BASE**	(“Periodontitis” OR "Periodontal Diseases" OR "Dental Scaling" OR "Debridement" OR "non-surgical periodontal therapy" OR "periodontal therapy" OR "periodontal treatment" OR "scaling and root planing" OR NSPT OR SRP OR PDT) AND (“type 2 diabetes" OR "type 2 diabetes mellitus" OR T2DM OR diabetes OR diabetic) AND ("Melatonin" OR melatonin OR "systemic melatonin" OR "oral melatonin" OR "melatonin supplementation" OR "melatonin therapy" OR "melatonin administration" OR "exogenous melatonin" OR "melatonin intake" OR "melatonin treatment" OR "melatonin use" OR "melatonin tablets" OR "melatonin pills")	85
**ProQuest**	NOFT((“Periodontitis” OR "Periodontal Diseases" OR "Dental Scaling" OR "Debridement" OR "non-surgical periodontal therapy" OR "periodontal therapy" OR "periodontal treatment" OR "scaling and root planing" OR NSPT OR SRP OR PDT) AND (“type 2 diabetes" OR "type 2 diabetes mellitus" OR T2DM OR diabetes OR diabetic) AND ("Melatonin" OR melatonin OR "systemic melatonin" OR "oral melatonin" OR "melatonin supplementation" OR "melatonin therapy" OR "melatonin administration" OR "exogenous melatonin" OR "melatonin intake" OR "melatonin treatment" OR "melatonin use" OR "melatonin tablets" OR "melatonin pills"))	6
**Google Scholar**	(“Periodontitis” OR "Periodontal Diseases" OR "Dental Scaling" OR "Debridement" OR "non-surgical periodontal therapy" OR "periodontal therapy" OR "periodontal treatment" OR "scaling and root planing" OR NSPT OR SRP OR PDT) AND (“type 2 diabetes" OR "type 2 diabetes mellitus" OR T2DM OR diabetes OR diabetic) AND ("Melatonin" OR melatonin OR "systemic melatonin" OR "oral melatonin" OR "melatonin supplementation" OR "melatonin therapy" OR "melatonin administration" OR "exogenous melatonin" OR "melatonin intake" OR "melatonin treatment" OR "melatonin use" OR "melatonin tablets" OR "melatonin pills")	100
**Research Gate**	non-surgical periodontal treatment, NSPT, melatonin, T2DM, diabetes	100

Table S2 Excluded articles and reasons for exclusion

Records identified from Electronic Databases	
Full-text articles excluded, with reasons (n=5)	
Author/Year	Reason for exclusion
Montero et al., 2017	1
El-Sharkawy et al., 2019	3
Tinto et al., 2020	3
Acatrinei et al., 2021	2
Navya et al., 2023	4
Pawane et al., 2023	2

<u>Reasons for exclusion:</u>

1 - Different melatonin administration protocol (n=1)

2 - Different intervention protocol (n=2)

3 - Ineligible population (n=2)

4 - Different study design (n=1)

Records identified from Grey Literature	
Full-text article excluded, with reason (n=1)	
Author/Year	Reason for exclusion
Ismail et al., 2021	1

<u>Reason for exclusion:</u>

1 - Ineligible population (n=1)

Table S3 Quality assessment of included studies

Outcome and follow-up	Patients (studies), N	Certainty	What happens
Change in PPD (pooled analysis): Follow up: 8 weeks	94 (2 RCTs)	⊕⊕◯◯ Low [a,d]	Systemic melatonin supplementation as an adjunct to NSPT resulted in a statistically significant reduction in PPD (SMD = −1.99, 95% CI: −2.54 to −1.43, $p < 0.00001$), with moderate heterogeneity ($I^2 = 48\%$). These findings were consistent across RCTs and further supported by an additional trial, comprising 55 patients, reporting significant PPD reductions at 3 and 6 months.
Change in CAL (pooled analysis): Follow up: 8 weeks	94 (2 RCTs)	⊕⊕◯◯ Low [a,d]	Systemic melatonin supplementation as an adjunct to NSPT resulted in a statistically significant improvement in CAL (SMD = −1.49, 95% CI: −2.03 to −0.95, $p < 0.00001$), with substantial heterogeneity ($I^2 = 69\%$), indicating variability in treatment effects across studies. These findings were consistent across RCTs and further supported by an additional trial, comprising 55 participants, reporting significant CAL improvements at 3 and 6 months.
Change in GCF MMP-8: Follow-up: up to 6 months	55 (1 RCT)	⊕⊕◯◯ Low [a,d]	In a single RCT including 55 participants, systemic melatonin supplementation as an adjunct to NSPT was associated with a greater reduction in GCF MMP-8 levels at 3 months compared with NSPT alone. Both groups showed reductions over time, but the intergroup difference favored melatonin only at the 3-month follow-up.
Change in GCF RANKL: Follow-up: up to 6 months	55 (1 RCT)	⊕⊕◯◯ Low [a,d]	In a single RCT including 55 participants, melatonin supplementation as an adjunct to NSPT was associated with a greater reduction in GCF RANKL levels at 3 months compared with NSPT alone. Although both groups demonstrated reductions at 3 and 6 months, the intergroup difference favored melatonin only at 3 months.
Change in GCF OPG: Follow-up: up to 6 months	55 (1 RCT)	⊕⊕◯◯ Low [a,d]	In a single RCT including 55 participants, no consistent benefit of adjunctive melatonin on GCF OPG levels was observed compared with NSPT alone. GCF OPG levels changed significantly only in the control group at 6 months, and the RANKL/OPG ratio remained similar between groups.
Change in HbA1c (pooled analysis): Follow up: 8 weeks	94 (2 RCTs)	⊕⊕◯◯ Low [a,d]	Systemic melatonin supplementation as an adjunct to NSPT resulted in a statistically significant improvement in HbA1c (SMD = −1.15, 95% CI: −1.63 to −0.68, $p < 0.00001$), with moderate heterogeneity ($I^2 = 38\%$).
Change in serum TAC (pooled analysis): Follow up: 8 weeks	94 (2 RCTs)	⊕⊕◯◯ Low [a,d]	Systemic melatonin supplementation as an adjunct to NSPT showed no statistically significant effect on serum TAC (SMD = 0.08, 95% CI: −0.05 to 0.20, $p = 0.23$), with considerable heterogeneity ($I^2 = 83\%$), indicating inconsistent findings across studies.
Change in serum hs-CRP (pooled analysis): Follow up: 8 weeks	94 (2 RCTs)	⊕⊕◯◯ Low [a,d]	Systemic melatonin supplementation as an adjunct to NSPT resulted in a statistically significant reduction in hs-CRP levels (SMD = −0.97, 95% CI: −1.22 to −0.73, $p < 0.00001$), with no observed heterogeneity ($I^2 = 0\%$), indicating a moderate and consistent effect across studies.

Outcome and follow-up	Patients (studies), N	Certainty	What happens
Change in saliva hs-CRP: Follow up: 8 weeks	55 (1 RCT)	⊕⊕◯◯ Low [a,d]	Systemic melatonin supplementation as an adjunct to NSPT resulted in a statistically significant reduction in saliva hs-CRP levels (from 2.73 ± 0.71 ng/mL to 1.24 ± 0.46 ng/mL, $p < 0.001$), with greater reductions observed in the melatonin-treated group.
Change in serum TNF-α (pooled analysis): Follow up: 8 weeks	94 (2 RCTs)	⊕⊕◯◯ Low [a,d]	Systemic melatonin supplementation as an adjunct to NSPT resulted in a non-significant reduction in TNF-α levels (SMD = −0.51, 95% CI: −1.92 to 0.90, $p = 0.48$), with no observed heterogeneity ($I^2 = 0\%$), indicating no consistent effect across studies.
Change in saliva TNF-α (pooled analysis): Follow up: 8 weeks	55 (1 RCT)	⊕⊕◯◯ Low [a,d]	Systemic melatonin supplementation as an adjunct to NSPT was associated with a non-significant reduction in saliva TNF-α levels, with no statistically significant differences observed between groups.
Change in serum IL-6 (pooled analysis): Follow up: 8 weeks	94 (2 RCTs)	⊕⊕◯◯ Low [a,d]	Systemic melatonin supplementation as an adjunct to NSPT resulted in a non-significant reduction in IL-6 levels (SMD = −5.04, 95% CI: −13.97 to 3.89, $p = 0.27$), with considerable heterogeneity ($I^2 = 99\%$), indicating substantial variability in treatment effects across studies. These findings were further supported by an additional trial, comprising 55 participants, reporting reductions at 3 and 6 months.
Change in saliva IL-6: Follow up: 8 weeks	55 (1 RCT)	⊕⊕◯◯ Low [a,d]	Systemic melatonin supplementation as an adjunct to NSPT resulted in a statistically significant reduction in saliva IL-6 levels (from 2.09 ± 0.60 ng/mL to 1.32 ± 0.32 ng/mL, $p < 0.001$), with greater reductions observed in the melatonin-treated group.
Change in serum IL-1β: Follow up: up to 6 months	99 (2 RCTs)	⊕⊕◯◯ Low [a,d]	Systemic melatonin supplementation as an adjunct to NSPT was associated with reductions in IL-1β levels across studies, with greater improvements observed in melatonin-treated groups, particularly at longer follow-up periods (3–6 months).
Change in serum MDA: Follow up: 8 weeks	44 (1 RCT)	⊕⊕◯◯ Low [a,d]	Systemic melatonin supplementation as an adjunct to NSPT resulted in a statistically significant reduction in serum MDA levels (from 17.2 ± 1.82 μM at baseline to 16.13 ± 1.76 μM post-treatment, $p < 0.001$), with greater reductions observed compared to the control group ($p = 0.008$).
Change in serum SOD: Follow up: 8 weeks	44 (1 RCT)	⊕⊕◯◯ Low [a,d]	Systemic melatonin supplementation as an adjunct to NSPT resulted in a statistically significant increase in serum SOD levels (from 13.91 ± 2.75 U/mL to 15.53 ± 4.37 U/mL, $p = 0.008$), with greater improvement compared to the control group ($p = 0.02$).
Change in serum GPx: Follow up: 8 weeks	44 (1 RCT)	⊕⊕◯◯ Low [a,d]	Systemic melatonin supplementation as an adjunct to NSPT resulted in a statistically significant increase in serum GPx levels (from 243.04 ± 68.37 U/mL to 262.04 ± 62.45 U/mL, $p = 0.004$), with greater improvement compared to the control group ($p = 0.04$).
Change in serum CAT: Follow up: 8 weeks	44 (1 RCT)	⊕⊕◯◯ Low [a,d]	Systemic melatonin supplementation as an adjunct to NSPT resulted in a statistically significant increase in serum CAT levels (from 24.23 ± 4.54 U/mL to 27.47 ± 4.12 U/mL, $p = 0.004$), with greater improvement compared to the control group ($p = 0.04$).

Outcome and follow-up	Patients (studies), N	Certainty	What happens
Change in serum TOS: Follow up: 8 weeks	50 (1 RCT)	⊕⊕◯◯ Low [a,d]	Systemic melatonin supplementation as an adjunct to NSPT resulted in a statistically significant reduction in serum TOS levels (from 0.016 ± 0.004 μmol at baseline to 0.01 ± 0.004 μmol post-treatment, $p < 0.05$), with greater reductions observed in the melatonin-treated group.
Change in saliva TOS: Follow up: 8 weeks	50 (1 RCT)	⊕⊕◯◯ Low [a,d]	Systemic melatonin supplementation as an adjunct to NSPT resulted in a statistically significant reduction in saliva TOS levels (from 0.073 ± 0.009 μmol to 0.031 ± 0.001 μmol, $p < 0.05$), with greater reductions observed in the melatonin-treated group.

Reasons for downgrading: a. risk of bias, b. inconsistency, c. indirectness, d. imprecision, e. publication bias
